# Opposing regulation of L-theanine biosynthesis by CsWRKY65 and CsWRKY69 in tea plant roots

**DOI:** 10.1093/hr/uhaf131

**Published:** 2025-05-21

**Authors:** Peiying Liu, Yongheng Zhang, Yumeng Bao, Dan Chen, Yezi Xiao, Hongyan Deng, Ziyao Ge, Pengjie Wang, Youben Yu

**Affiliations:** College of Horticulture, Northwest A&F University, Yangling, Shaanxi 712100, China; National Key Laboratory for Tea Plant Germplasm Innovation and Resource Utilization, Key laboratory of Biology, Genetics and Breeding of Special Economic Animals and Plants, Ministry of Agriculture and Rural Affairs, National Center for Tea Plant Improvement, Tea Research Institute, Chinese Academy of Agricultural Sciences, Hangzhou 310008, China; College of Horticulture, Northwest A&F University, Yangling, Shaanxi 712100, China; College of Horticulture, Northwest A&F University, Yangling, Shaanxi 712100, China; College of Horticulture, Northwest A&F University, Yangling, Shaanxi 712100, China; College of Horticulture, Northwest A&F University, Yangling, Shaanxi 712100, China; College of Horticulture, Northwest A&F University, Yangling, Shaanxi 712100, China; College of Horticulture, Northwest A&F University, Yangling, Shaanxi 712100, China; College of Horticulture, Northwest A&F University, Yangling, Shaanxi 712100, China

Dear Editor,

 L-theanine is a unique nonproteinaceous amino acid that confers umami taste to tea infusions. In addition to its taste-enhancing properties, l-theanine demonstrates the ability to provide a variety of health benefits, including antioxidant activities, cardiovascular protection, anticancer, antianxiety, and immune regulatory [1]. Therefore, targeted modulation of l-theanine biosynthesis in tea plants offers a promising approach for enhancing both the sensory characteristics and health benefits of tea products. The biosynthesis of l-theanine in tea plants primarily occurs in roots through the coordinated action of alanine decarboxylase (AlaDC) and l-theanine synthetase (TSI). Therein, CsAlaDC is responsible for catalyzing the production of ethylamine, whereas CsTSI directly catalyzes the conversion of ethylamine and glutamate into l-theanine. The inability of other plants to synthesize l-theanine is primarily attributed to ethylamine deficiency [2]. Notably, the level of ethylamine, rather than the expression level of *CsTSI*, predominantly determines l-theanine content in tea roots. These observations underscore the pivotal role of *CsAlaDC* in regulating l-theanine levels in tea plants [2].

Recently, several transcription factors (TFs), including CsMYB40, CsHHO3, and CsGATA17, have been implicated in modulating the expression of *CsAlaDC* and, consequently, l-theanine content [3, 4]. Collectively, these findings indicate that l-theanine synthesis in tea plants is governed by a complex regulatory network. The identification of additional regulators is crucial for elucidating the intricate mechanisms underlying l-theanine accumulation. However, whether WRKY TFs are involved in the regulation of l-theanine biosynthesis in tea roots remains to be investigated. To this end, we investigated the *cis-*elements of the *CsAlaDC* promoter and found that multiple W-box elements were present. This suggests that WRKY members may play a role in regulating *CsAlaDC* expression (Fig. 1A). Subsequently, we analyzed the expression levels of 33 WRKY members across distinct tissues and found that 10 members, including *CSS0019338*, *CSS0005905*, *CSS0009850*, *CSS0001726*, *CSS0024751*, *CSS0024614*, *CSS0024883*, *CSS0021156*, *CSS0028565*, and *CSS0013701*, exhibited high expression levels in roots and were highly correlated with *CsAlaDC* (Pearson correlation coefficient >0.9) (Fig. 1B). Therefore, these 10 WRKY members were selected to verify their regulatory effects on the expression of *CsAlaDC* using dual-LUC reporter assays. As indicated in Fig. 1C, compared to the control group, the expression of CSS0024614 (CsWRKY65) significantly enhanced LUC signaling driven by the *CsAlaDC* promoter, whereas the expression of CSS0009850 (CsWRKY69) significantly reduced it. Among the remaining WRKY members tested, none exhibited a significant regulatory effect. These results demonstrated that CsWRKY69 represses, whereas CsWRKY65 activates, the expression of *CsAlaDC in planta*. To determine the subcellular localization of CsWRKY65 and CsWRKY69, transient expression experiments were conducted in tobacco leaves. The green fluorescence signals of CsWRKY65-GFP and CsWRKY69-GFP were clearly observed in the nucleus, consistent with their roles as TFs (Fig. 1D).

**Figure 1:**
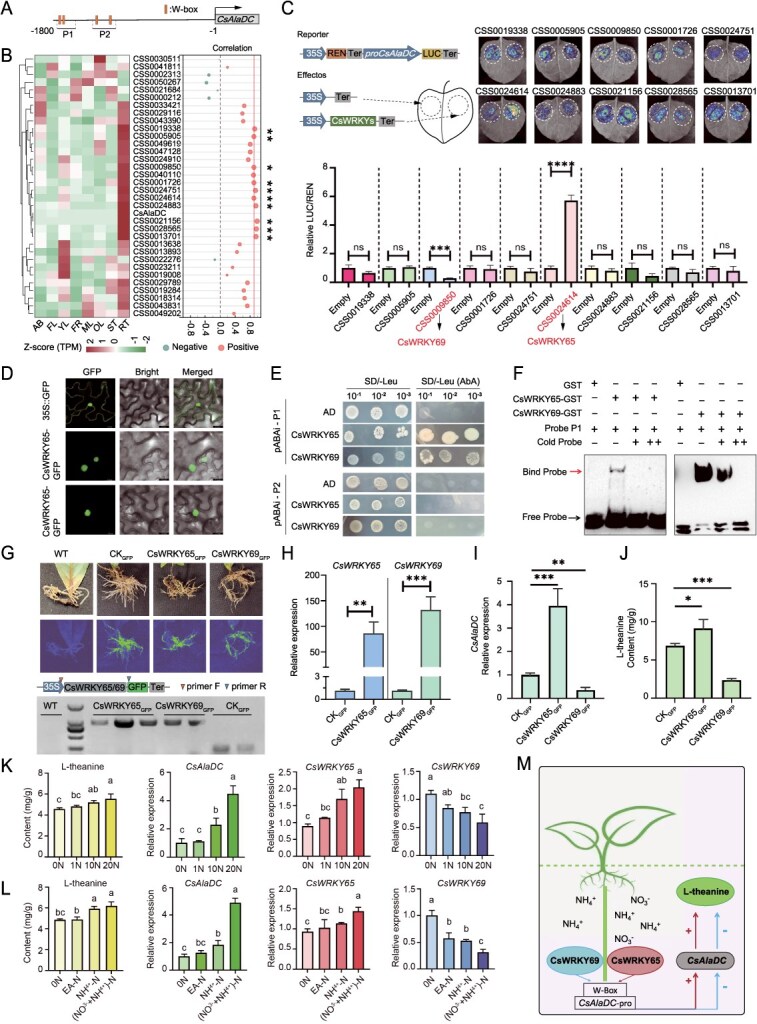
CsWRKY65 and CsWRKY69 play opposite regulatory roles in *CsAlaDC* expression to regulate l-theanine biosynthesis in tea plants. (A) The WRKY TF binding sites on the *CsAlaDC* promoter. (B) Correlation analysis conducted on the expression levels of WRKY family and *CsAlaDC* in various parts of tea. (C) Measurement of the LUC/REN ratio in different groups of tobacco leaves. Data are representative of the mean ± SD of three independent experiments. Significant differences were determined by the Student’s *t* test (^****^P ≤ 0.0001, ^***^P ≤ 0.001). (D) Subcellular localization of 35S::*CsWRKY65/69*-GFP in tobacco cells. (E) In the yeast single hybrid experiment, the experimental group grew normally on SD/−Ura/−Leu deficient plates containing 100 ng/ml AbA. (F) Purified protein was mixed with probes (WRKY Site 1) for EMSA. The presence (+) or absence (−) is shown. The biotin probe contained a 3′ biotin label, and the cold probe lacked the 3′ biotin label. (G) *CsWRKY65* and *CsWRKY69* stably overexpressed in tea roots. Transgenic roots were identified based on GFP (green fluorescent protein) and regular polymerase chain reaction (PCR). ‘WT’ represents the wild-type of *Camellia sinensis* var.‘Longjing Changye’. ‘CK_GFP_’ represents transgenic explants containing 35S::GFP binary plasmid; ‘CsWRKY65_GFP_’ represents transgenic explants containing 35S::*WRKY65*-GFP binary plasmid; ‘CsWRKY69_GFP_’ represents transgenic explants containing 35S::*WRKY69*-GFP binary plasmid. (H) The expression level of *CsWRKY65* and *CsWRKY69* in transgenic hairy roots. (I) The expression level of *CsAlaDC* in transgenic hairy roots. (J) l-Theanine content in transgenic plants. In H–J, data are representative of the mean ± SD of three independent experiments. Significant differences were determined by the Student’s *t* test (^***^P ≤ 0.001, ^**^P ≤ 0.01, ^*^P ≤ 0.05). (K) Analysis of gene expression pattern and determination of l-theanine content under different concentrations of N treatments. (L) Analysis of gene expression pattern and determination of l-theanine content under different forms of N treatments. In K and L, data are representative of the mean ± SD of three independent experiments. Significant differences were determined by the Tukey multiple comparison test. (M) Proposed model for the transcriptional regulation of CsWRKY65 and CsWRKY69 regulating l-theanine synthesis by directly binding to the promoter of *CsAlaDC* in response to nitrogen fluctuations.

To investigate whether CsWRKY65 and CsWRKY69 directly bind to the *CsAlaDC* promoter, two promoter fragments, P1 (−1726 to −1517) and P2 (−1332 to −1045), each containing two W-box *cis-*elements, were utilized in yeast one-hybrid (Y1H) assays. The results indicated that yeast cells grew well on selective medium when AD-CsWRKY65 was transformed into CsAlaDC-P1 bait cells but failed to proliferate when transformed into CsAlaDC-P2 bait cells. Similar results were observed with AD-CsWRKY69 (Fig. 1E). To further validate this binding, electrophoretic mobility shift assays (EMSAs) were conducted. The results demonstrated that band shifts occurred upon incubation of GST-WRKY65 or GST-WRKY69 with a biotin-labeled probe, and these shifts were attenuated with increasing concentrations of unlabeled cold probe (Fig. 1F). These results confirm that both CsWRKY65 and CsWRKY69 directly bind to the P1 region of the *CsAlaDC* promoter.

To further investigate the roles of CsWRKY65 and CsWRKY69 in regulating *CsAlaDC* expression and l-theanine in tea plants, we overexpressed these TFs in the roots of tea plants by *Agrobacterium rhizogenes* strains of ATCC15834 according to the method described by Ma *et al.* [5] (Fig. 1G). The results demonstrated that *CsWRKY65* and *CsWRKY69* were successfully overexpressed 83.29 times and 130.10 times, respectively, in tea roots (Fig. 1H). Notably, an increase in *CsAlaDC* expression and l-theanine content was observed in *CsWRKY65*-overexpressed roots (Fig. 1I and J). Conversely, overexpression of *CsWRKY69* resulted in a decrease in both *CsAlaDC* expression and l-theanine content (Fig. 1I and J). These findings further support the conclusion that CsWRKY65 and CsWRKY69 play opposite roles in l-theanine accumulation by activating and repressing *CsAlaDC* expression in tea roots, respectively.

Nitrogen (N) supply is an important factor influencing l-theanine content. In order to explore the regulatory role of CsWRKY65 and CsWRKY69 in l-theanine accumulation in response to various concentrations and forms of N, 2-year-old tea seedlings were cultivated in hydroponic solution with various concentrations (0, 1, 10, or 20 N; 1 N was 0.72 mM NO_3_^−^ -N plus NH_4_^+^ -N) and forms (0 N, EA-N [1.43 mM], NH_4_^+^ -N [1.43 mM], or NO_3_^−^ -N plus NH_4_^+^ -N [1.43 mM]) of N for 10 and 18 days, respectively, to assess changes in l-theanine levels and relative gene expression levels. The results demonstrated that the increase of N concentration significantly promoted the accumulation of l-theanine, accompanied by a concurrent elevation in *CsAlaDC* and *CsWRKY65* expression, whereas it caused a decrease in the expression of *CsWRKY69* (Fig. 1K). Similarly, compared to the application of a single form of N, the combined application of NO_3_^−^ -N and NH_4_^+^ -N could promote the accumulation of l-theanine and the expression levels of *CsAlaDC* and *CsWRKY65* while repressing the expression of *CsWRKY69* (Fig. 1K). These findings indicated that an increased supply of N potentiated the stimulatory role of CsWRKY65 in l-theanine accumulation while concurrently weakening the inhibitory role of CsWRKY69, resulting in an increase in l-theanine content. Importantly, this observation contrasts with previously identified activators (CsMY40) and repressors (CsHHO3) of l-theanine biosynthesis [3], underscoring the complexity of l-theanine biosynthesis regulation.

In summary, our study demonstrates that CsWRKY65 and CsWRKY69 can directly bind to the promoter of *CsAlaDC* and regulate its expression, thereby exerting opposite regulatory effects on l-theanine accumulation. Furthermore, under varying nitrogen levels, the expression of these TFs follows opposite trends, which in turn coordinates the regulation of l-theanine levels in tea plant (Fig. 1L). These findings provide a crucial theoretical foundation for exploring the regulatory factors of l-theanine synthesis and offers valuable insights for tea plant breeding.

## Data Availability

The data that support the findings of this study are publicly available in the Tea Plant Information Archive (TPIA): a comprehensive knowledge database for tea plant (http://tpia.teaplants.cn/index.html).

## References

[1] Li M, Liu H, Wu D. et al. l-Theanine: a unique functional amino acid in tea (*Camellia sinensis* L.) with multiple health benefits and food applications. Front Nutr. 2022;9:853846 35445053 10.3389/fnut.2022.853846 PMC9014247

[2] Zhu B, Guo J, Dong C. et al. *CsAlaDC* and *CsTSI* work coordinately to determine theanine biosynthesis in tea plants (*Camellia sinensis* L.) and confer high levels of theanine accumulation in a non-tea plant. Plant Biotechnol J. 2021;19:2395–7 34626137 10.1111/pbi.13722 PMC8633503

[3] Guo J, Zhu B, Chen Y. et al. Potential ‘accelerator’ and ‘brake’ regulation of theanine biosynthesis in tea plant. Hortic Res. 2022;9:uhac169 36324642 10.1093/hr/uhac169 PMC9614919

[4] Gao Y, Sun C, Zhang X. et al. Identification of the GATA transcription factor family in tea plant (*Camellia sinensis*) and the characterizations in nitrogen metabolism. Plant Physiol Biochem. 2025;221:109661 39987619 10.1016/j.plaphy.2025.109661

[5] Ma J, Liu N, Sun X. et al. Establishment of an efficient transformation system and its application in regulatory mechanism analysis of biological macromolecules in tea plants. Int J Biol Macromol. 2023;244:125372 37321436 10.1016/j.ijbiomac.2023.125372

